# Excessive antibiotics use increased in-hospital mortality in intracerebral hemorrhage patients with stroke-associated infection

**DOI:** 10.1038/s41598-020-58772-3

**Published:** 2020-02-06

**Authors:** Fei Wang, Yinghua Wang, Junfeng Shi, Lijuan Shen, Xinyu Xu, Ting-ting Jiang, Li Wang, Qing Chang, Shanyou Hu, Xiao Wu

**Affiliations:** 1 Emergency Department, Jiading District Central Hospital Affiliated Shanghai University of Medicine & Health Sciences, Shanghai, China; 2 Department of Pharmacy, Jiading District Central Hospital Affiliated Shanghai University of Medicine & Health Sciences, Shanghai, China; 3 Shanghai Key Laboratory for Molecular Imaging, Shanghai University of Medicine & Health Sciences, Shanghai, China; 4 Department of Clinical Laboratory, Jiading District Central Hospital Affiliated Shanghai University of Medicine & Health Sciences, Shanghai, China; 5 Jiading Town Community Healthcare Center of Jiading District, Shanghai, China; 6 Department of Critical Care Medicine, Jiading District Central Hospital Affiliated Shanghai University of Medicine & Health Sciences, Shanghai, China; 7 Clinical Research Center, Jiading District Central Hospital Affiliated Shanghai University of Medicine & Health Sciences, Shanghai, China

**Keywords:** Stroke, Stroke

## Abstract

Intracerebral hemorrhage (ICH) is associated with higher incidence of stroke-associated infection (SAI) as well as antibiotic use. However, there were few methods for judging proper antibiotic use in clinical manner. We introduce an index of antibiotic use, called personal antibiotic use density (PAUD), to evaluate the relation between antibiotic use and prognosis of ICH patients with SAI. A total of 162 in 570 ICH patients were observed to diagnose as SAI. Comparing with the survival patients, PAUD, ICH volume, National Institutes of Health Stroke Scale (NIHSS) score and ICH score were significantly higher among those who died, while the Glasgow Coma Scale score and the length of stay were significantly lower (P < 0.05). PAUD was identified as an independent risk factor of in-hospital death (OR 2.396, 95% CI 1.412–4.067, P = 0.001). In-hospital mortality was significantly lower in the low (P = 0.027) and intermediate PAUD (P < 0.001) groups than that in the high PAUD group. Cumulative in-hospital survival was significantly higher in low and intermediate PAUD groups (log rank test, P < 0.001). PAUD correlated positively with NIHSS score (r = 0.224, P < 0.001) and in-hospital mortality (r = 0.268, P = 0.001). The study indicated that PAUD is closely related to in-hospital prognosis of ICH patients with SAI. Higher PAUD may not be associated with better prognosis, but instead, higher risk of death.

## Introduction

Stroke-associated infection (SAI)^1^, also known as post-stroke infection, is defined as the absence of signs of infection at stroke and during infection latency, followed by infection within the first week after stroke^2^. SAIs, such as pulmonary and urinary tract infections, are important complications of acute stroke. They complicate the disease and can worsen prognosis^3,4^. In the clinical treatment of this type of patients, the use of antibiotics is extremely common, but adverse reactions^5^ and antibiotic abuse can damage important organs and give rise to resistance, making treatment more difficult^6^. Therefore, evaluation of antibiotic use is particularly important.

Antibiotic use density (AUD) is a group- or population-level index that refers to the ratio of antibiotic consumption to the number of days that patients are hospitalized^7^. AUD is often used in clinical economics^8^. We propose here the concept of personal AUD (PAUD) to assess antibiotic use at the individual level. PAUD may better capture patients’ use of antibiotics during hospitalization and the daily antibiotic load. It may be useful for evaluation of personal antibiotic use.

Intracerebral hemorrhage is associated with higher mortality than other types of stroke^9^ as well as higher incidence of SAI^10^ and more common antibiotic use^11^. The potential relationship between the use of antibiotics and prognosis of intracerebral hemorrhage patients with SAI is unclear. The purpose of the present study was to examine the potential association between PAUD and prognosis in intracerebral hemorrhage patients with SAI.

## Methods

### Patients

We continuously collected data on patients with intracerebral hemorrhage who were admitted to our hospital from January 2015 to September 2017. All patients were diagnosed according to the AHA/ASA Guidelines for the Management of Spontaneous Intracerebral Hemorrhage^12^. Patients were excluded if they had (1) recurrent cerebral hemorrhage; (2) anticoagulant drug-related cerebral hemorrhage; (3) patients who were bedridden during the last three months, regardless of reason; (4) history of mental illness.

The excluded conditions in patients with infection were that (1) infection occurs one week after stroke (n = 9); (2) confirmed infection before admission (n = 5); (3) severe hepatic/renal insufficiency (n = 3); (4) abandon treatment within 48 hours (n = 2).

The study protocol was approved by the Ethics Review Board of Jiading District Central Hospital (2017-ZD-03). Written informed consent was waived by the Ethics Review Board due to the retrospective nature of the study. Patient data were anonymized. The procedures followed were in accordance with the standards of the ethics committee of Jiading District Central Hospital and with the Declaration of Helsinki (1975, revised 2013).

### Diagnostic criteria

SAI was defined as infection occurring during the first week after stroke^2^. Stroke-associated pneumonia was defined as the presence of relevant clinical signs and/or symptoms (such as cough and sputum, unilateral inhalation moist rale, and bronchial breath sound), together with at least one of the following auxiliary examination results: leukocytosis, fever or positive chest X-ray^13^. Stroke-associated urinary tract infection was defined as the existence of relevant clinical symptoms and/or signs (such as difficulty in urination and frequent urination) combined with positive microbial culture results or fever (≥37.5 °C), or negative microbial culture results with leukocytosis (>11 × 10^9^/L)^13^.

### Data collection

We retrospectively analyzed general demographic data (including gender, age), past medical history [including hypertension, diabetes, chronic obstructive pulmonary disease (COPD)], hematoma site (above or under the curtain), hematoma volume, rate of combined intraventricular hemorrhage and length of stay. Also recorded were the National Institute of Health Stroke Scale (NIHSS) score, Glasgow Coma Scale (GCS) score, as well as infection-related laboratory parameters before antibiotic use, including white blood cell count (WBC), neutrophil-to-lymphocyte ratio (NLR), high sensitivity C-reactive protein (hs-CRP), procalcitonin (PCT) and blood urea nitrogen (BUN). The intracerebral hemorrhage scale (ICH) score^14^ was calculated and recorded according to each patient’s admission status and cranial computed tomography.

### Group definitions

The used antibiotics were included Cephalosporins (Cefuroxime, Ceftazidime and Cefepime), Carbapenems (Imipenem and Cilastatin Sodium, Meropenem), Quinolones (Levofloxacin, Moxifloxacin), Azithromycin, Piperacillin and tazobactam sodium and Amikacin. PAUD was defined as cumulative personal defined daily dose (DDD)^15^ divided by the hospital stay (in days). In other words, PAUD = Individual antimicrobial consumption during patient hospitalization/Length of stay. The individual antimicrobial consumption during patient hospitalization = ((antibiotic A) specifications * daily administration frequency * Duration of antibiotic use)/the standard DDD of (antibiotic A) + ((antibiotic B) specifications * daily administration frequency *Duration of antibiotic use)/the standard DDD of (antibiotic B) + …. The standard DDD of antibiotics were confirmed on the website of World Health Organization (WHO). The website is https://www.whocc.no/atc_ddd_index. Patients were divided into a survival group (n = 111) and death group (n = 51). Patients were also divided according to PAUD tertiles into a low PAUD group (<0.38, n = 53), intermediate PAUD group (0.38–0.75, n = 55) and high PAUD group ≥0.75, n = 54).

### Statistical analysis

Statistical analysis was performed using SPSS 19.0 (IBM, Chicago, IL, USA). Normally distributed data were expressed as mean ± SD and compared between groups using Student’s *t* test. Non-normally distributed data were expressed as median (interquartile range, IQR) and compared between groups using the non-parametric Mann-Whitney test. Numerical data were expressed as frequencies and compared between groups using the χ^2^ test. Kaplan-Meier survival analysis was carried out, and survival was counted from the date of admission until all-cause death during hospitalization. Multivariate Cox regression was used to identify factors associated with in-hospital mortality. Spearman correlation was used to analyze relationships between PAUD and other variables. P < 0.05 was considered statistically significant.

## Results

### General patient characteristics

**Figure 1:**
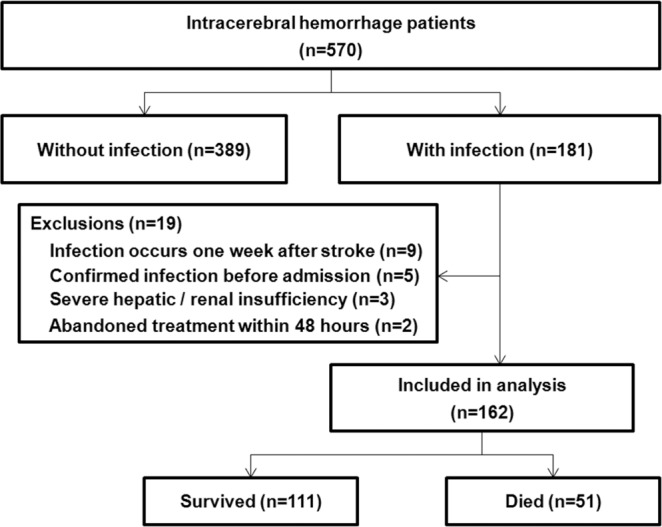
Flow diagram of patients in the study.

We continuously collected data on 570 patients with intracerebral hemorrhage from January 2015 to September 2017. The in-hospital mortality rate was 15.6% (89/570) and the SAI rate was 28.4% (162/570). Subsequent analysis was performed only on the 162 cases with SAI (Fig. 1), of whom 111 (68.5%) were men. Mean age was 66.6 ± 15.2 years. Of all 162 patients, 50 were admitted to the ICU, accounting for 30.9%. The most frequent infection was pneumonia [83.9% (136/162)]. In-hospital mortality was significantly higher among intracerebral hemorrhage patients with SAI [31.5% (51/162)] than among all 570 patients examined during the study period [15.6% (89/570), P < 0.001].

### Comparison of clinical factors and outcomes among patients stratified by prognosis or PAUD

The death group showed significantly higher values than the survival group for the following clinical indices: ICH volume [43.3 (23.8, 68.4) *vs*. 17.9 (7.15, 41.1), P < 0.001], NIHSS score [22 (17, 25) *vs*. 11 (4, 18), P < 0.001], ICH score [3 (2, 4) *vs*. 1 (1, 2), P < 0.001], WBC [13.9 (10.8, 16.8) *vs*. 9.6 (7.2, 13.1), P < 0.001], NLR [15.1 (4.6, 20.2) *vs*. 8.5 (4.5, 13.8), P = 0.013], the rate of ICU admitted (43.1% vs. 25.2%, P = 0.022) and PAUD [0.83 (0.33, 1.12) *vs*. 0.47 (0.26, 0.73), P = 0.001]. Conversely, the death group showed significantly lower values on the following clinical indices: GCS score [5 (3, 12) *vs*. 14 (8, 15), P < 0.001], personal accumulated consumption of antibiotics in DDDs [2.4 (1.0, 5.9) *vs*. 11.7 (6.0, 17.5), P < 0.001], the time of infections start (2.8 ± 1.8 vs. 4.1 ± 1.7, P < 0.001) and length of hospital stay [6 (4, 17) *vs*. 25 (18, 32), P < 0.001]. The death and survival groups did not differ significantly in other factors (Table 1).

**Table 1:** Clinical characteristics of patients who survived or died in hospital.

	Survived (n = 111)	Died (n = 51)	Statistical analysis	*P*
Demographics				
Male, n (%)	74 (66.7)	37 (72.5)	*x*^2^ = 0.561	0.454
Age in yr, mean ± sd	67.7 ± 15.0	64.2 ± 15.7	t = 1.394	0.165
Age ≥80 yr, n (%)	29 (26.1)	13 (25.5)	*x*^2^ = 0.007	0.932
Comorbidities, n (%)				
Hypertension	88 (79.3)	37 (72.5)	*x*^2^ = 0.898	0.343
Diabetes	14 (12.6)	9 (17.6)	*x*^2^ = 0.727	0.394
COPD	2 (1.8)	0 (0.0)	*x*^2^ = 0.039	0.843
Site of infection				
Lung, n (%)	93 (83.8)	43 (84.3)	-	0.222
Urinary system, n (%)	6 (5.4)	0 (0.0)		
Other, n (%)	12 (10.8)	8 (15.7)		
Site of bleeding, n (%)				
Supratentorial	106 (95.5)	44 (86.3)	*x*^2^ = 3.092	0.079
Infratentorial	5 (4.5)	7 (13.7)		
Site of bleeding, n (%)				
Basal ganglia	57 (51.4)	28 (54.9)	z = −0.362	0.718
Brain lobe	30 (27.0)	8 (15.7)		
Thalamus	12 (10.8)	4 (7.8)		
Cerebellum	4 (3.6)	2 (3.9)		
Ventricle	7 (6.3)	5 (9.8)		
Brainstem	1 (0.9)	4 (7.8)		
ICH volume in ml, median (P25, P75)	17.9 (7.15, 41.1)	43.3 (23.8, 68.4)	z = −4.222	<0.001
ICH volume ≥ 30 ml, n (%)	41 (36.9)	36 (70.6)	*x*^2^ = 15.867	<0.001
Concurrent ventricular hemorrhage, n (%)	47 (42.3)	28 (54.9)	*x*^2^ = 2.217	0.136
NIHSS score, median (P25, P75)	11 (4, 18)	22 (17, 25)	z = −6.236	<0.001
GCS score, median (P25, P75)	14 (8, 15)	5 (3, 12)	z = −6.002	<0.001
ICH score, median (P25, P75)	1 (1, 2)	3 (2, 4)	z = −5.150	<0.001
ICU admitted, n (%)	28 (25.2)	22 (43.1)	*x*^2^ = 5.254	0.022
Time of infections start, mean ± sd	4.1 ± 1.7	2.8 ± 1.8	t = 4.594	<0.001
Personal accumulated consumption of antibiotics in DDDs, median (P25, P75)	11.7 (6.0, 17.5)	2.4 (1.0, 5.9)	z = −5.877	<0.001
length of stay in days, median (P25, P75)	25 (18, 32)	6 (4, 17)	z = −6.976	<0.001
PAUD in per-day, median (P25, P75)	0.47 (0.26, 0.73)	0.83 (0.33, 1.12)	z = −3.397	0.001
Infection-related laboratory parameters before antibiotic use, median (P25, P75)				
WBC in 10^9^/L	9.6 (7.2, 13.1)	13.9 (10.8, 16.8)	z = −4.395	<0.001
NLR	8.5 (4.5, 13.8)	15.1 (4.6, 20.2)	z = −2.495	0.013
hs-CRP in mg/L	18 (8, 45)	29.5 (7, 88)	z = −1.069	0.285
PCT in ug/L	0.11 (0.06, 0.48)	0.19 (0.1, 1.94)	z = −1.557	0.120
BUN in mmol/L	5.9 (4.5, 8.1)	6.6 (5.2, 10.5)	z = −1.634	0.102

Abbreviations: COPD: chronic obstructive pulmonary disease; ICH: intracranial hemorrhage; NIHSS: national institute of health stroke scale; GCS: glasgow coma scale; DDD: defined daily dose; PAUD: personal antibiotic use density; WBC: white blood cell count; NLR: neutrophil-to-lymphocyte ratio; hs-CRP: high sensitivity C-reactive protein: PCT: procalcitonin; BUN: blood urea nitrogen.

**Figure 2:**
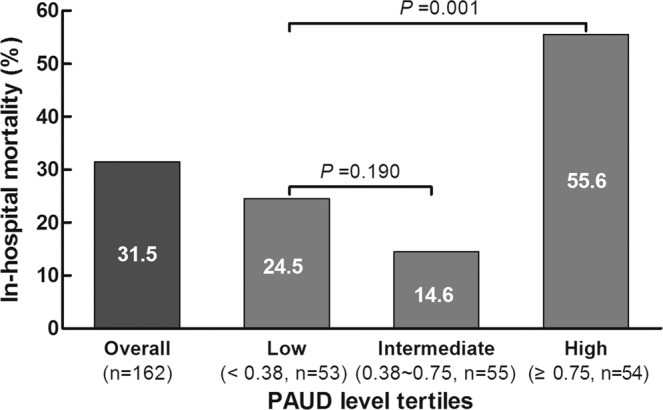
Correlation between in-hospital mortality and PAUD in patients stratified by PAUD tertile (low, intermediate, high). PAUD values in each group are shown below the name of each group.

In-hospital mortality was significantly lower in the low PAUD group (24.5%) and intermediate PAUD group (14.6%) than in the high PAUD group (55.6%; P < 0.001; Fig. 2).

### Risk factors for in-hospital death

Multivariate Cox regression was conducted with adjustment for sex, age, WBC, ICH volume, infratentorial, concurrent ventricular hemorrhage and GCS score. The results identified three risk factors for in-hospital death (Model 1, Table 2): PAUD (OR 2.396, 95% CI 1.412–4.067, P = 0.001), NIHSS score (OR 1.012, 95% CI 1.067–1.139, P < 0.001) and NLR (OR 1.040, 95% CI 1.006–1.074, P = 0.021). Risk of in-hospital mortality was significantly lower in the low PAUD group than in the high PAUD group (OR 0.451, 95% CI 0.222–0.913, P = 0.027), and it was lower in the intermediate PAUD group than in the high PAUD group (OR 0.211, 95% CI 0.090–0.493, P < 0.001; Model 2, Table 2). After adjustment for sex, age, WBC and ICH score, Model 3 identified three independent risk factors of in-hospital mortality: PAUD (OR 2.952, 95% CI 1.711–5.094, P < 0.001), NIHSS score (OR 1.095, 95% CI 1.057–1.134, P < 0.001) and NLR (OR 1.038, 95% CI 1.004–1.074, P = 0.027; Table 2).

**Table 2:** Multivariate Cox regression to identify potential factors associated with in-hospital mortality.

Model	Factor	Adjusted		
		OR	P	95% CI
1	PAUD	2.396	0.001	1.412, 4.067
	NIHSS score	1.102	<0.001	1.067, 1.139
	NLR	1.040	0.021	1.006, 1.074
2	PAUD < 0.38	0.451	0.027	0.222, 0.913
	0.38–0.75	0.211	<0.001	0.090, 0.493
	≥0.75	Ref.		
	NIHSS score	1.101	<0.001	1.065, 1.138
	NLR	1.039	0.026	1.005, 1.076
3	PAUD	2.952	<0.001	1.711, 5.094
	NIHSS score	1.095	<0.001	1.057, 1.134
	NLR	1.038	0.027	1.004, 1.074

Model 1 and 2 adjusted: sex, age, WBC, ICH volume, infratentorial, concurrent ventricular hemorrhage and GCS score.

Model 3 adjusted: sex, age, WBC and ICH score.

Abbreviations: PAUD: personal antibiotic use density; NIHSS: national institute of health stroke scale; NLR: neutrophil-to-lymphocyte ratio; WBC: white blood cell count; ICH: intracranial hemorrhage; GCS: glasgow coma scale.

### PAUD tertile and survival

**Figure 3:**
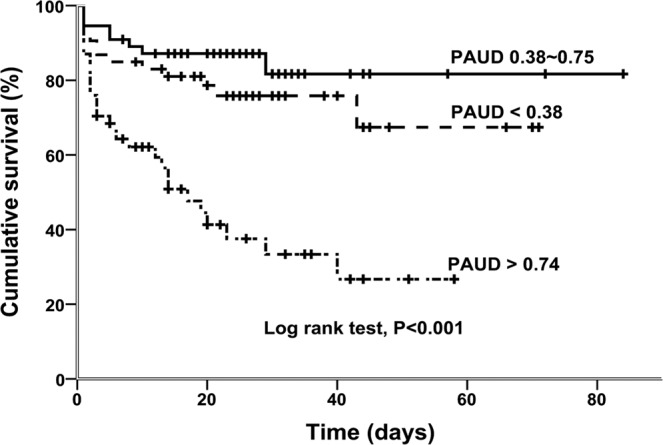
Kaplan-Meier curve showing in-hospital mortality in patients stratified by PAUD tertiles.

Cumulative in-hospital survival was significantly higher in the low and intermediate PAUD groups than in the high PAUD group (log rank test, P < 0.001; Fig. 3).

### PAUD and clinical factors or prognosis

Spearman correlation analysis showed that PAUD correlated positively with NIHSS score (r = 0.216, P = 0.006) and in-hospital mortality (r = 0.268, P = 0.001; Table 3).

**Table 3:** Correlation of PAUD with clinical features in patients.

Factor	PAUD	
	Spearman’s correlation coefficient	p
Male gender	−0.143	0.069
Age	0.081	0.307
ICH volume	0.018	0.819
NIHSS score	0.216	**0.006**
GCS score	−0.103	0.192
Infratentorial	−0.088	0.266
Concurrent ventricular hemorrhage	0.147	0.062
WBC	0.137	0.083
NLR	0.021	0.794
In-hospital death	0.268	**0.001**

Abbreviations: PAUD: personal antibiotic use density; ICH: intracranial hemorrhage; NIHSS: national institute of health stroke scale; GCS: glasgow coma scale; WBC: white blood cell count; NLR: neutrophil-to-lymphocyte ratio.

## Discussion

This is the first study to propose the concept of PAUD and to use it to evaluate clinical prognosis. In this study, the incidence of SAI, due mainly to pulmonary infection, was relatively high among patients with intracerebral hemorrhage. Among these patients, SAI was associated with higher in-hospital mortality. Our results suggests that PAUD is closely related to in-hospital prognosis of intracerebral hemorrhage patients with SAI. Higher PAUD may be associated with greater risk of death.

Our study suggests that the incidence of SAI is nearly 30% among intracerebral hemorrhage patients, and that mortality is as high as 31.5% among such patients. These results are consistent with previous work^16,17^. Unfortunately, prophylactic use of antibiotics by patients with stroke does not appear to reduce occurrence of SAI^18^. This highlights the need to evaluate the use of antibiotics among such patients.

AUD, widely used in management and research fields, can be used to compare antibiotic use across time periods and geographic regions and thereby guide strategies for rational, cost-effective antibiotic use^19–21^. However, AUD looks at aggregate antibiotic use by populations, which can mask clinically important differences among patients and limit our ability to assess safety and efficacy of specific dosing regimens of specific antimicrobial agents. Therefore, the present study proposes the concept of PAUD. Our study explored the relationship between PAUD and prognosis in intracerebral hemorrhage patients with SAI, in order to provide new approaches to evaluate antibiotic use in this vulnerable patient group.

For these patients in our study, in-hospital mortality was associated with significantly lower individual cumulative DDD and shorter length of stay, as well as higher bleeding volume, rate of combined intraventricular hemorrhage and lower GCS scores. These results likely reflect the fact that in-hospital mortality shortens the length of stay, which in turn reduces the individual cumulative DDD. However, at the same time, in-hospital mortality was associated with significantly higher PAUD. In other words, although the patients who died were hospitalized for shorter periods than those who survived, they had a much higher daily average cumulative personal defined daily dose. This result suggests that antibiotic use may be related to prognosis. Indeed, we found PAUD to be an independent risk factor of in-hospital death among these patients. For each unit increase in PAUD, risk of death increased 1.4-fold.

Patients with SAI may require a different approach to antibiotic use than other patients with infectious diseases. The broad-spectrum combination therapies typically applied to patients with infectious diseases, especially critically ill patients, may damage the normal microbial flora in patients with SAI, compromising their ability to combat infection and thereby increasing risk of mortality^22^. In addition, intracerebral hemorrhage patients with SAI have a neurological deficit not found in patients with primary infectious diseases. This may call for adjustment of the types and amounts of antibiotics to optimize therapy to individual patients and ensure rational antibiotic use.

In the present study, we found NLR to be associated with poor short-term survival, confirming previous results from our research group^23–25^. We found that NIHSS score was an independent risk factor of death, and that it correlated positively with PAUD. This indicates that the severity of neurological deficits is closely related to the use of antibiotics: the loss of swallowing function^26^, hypostatic pneumonia caused by impairment of physical activity, and aspiration or reflux caused by disturbance of consciousness^27^ may lead to infection, in turn increasing PAUD. Our findings support previous results linking NIHSS score to the developed infection of patients with stroke^28^, which may help explain the observed association between high PAUD and in-hospital mortality. However, we did not observe an association of hematoma volume or GCS score with PAUD, although both factors are closely related to the occurrence of SAI after intracerebral hemorrhage^29–31^. It may be that the NIHSS score more comprehensively reflects neurological impairment due to hematoma volume and impaired consciousness captured in the GCS score. In any event, our findings and the literature highlight the need for clinicians to pay sufficient attention to patients with intracerebral hemorrhage, regardless of the severity of their neurological deficits, including timely assessment of swallowing function^32^, retention of the gastric tube^33^ and effective counseling and other interventions to help patient’s family members prevent SAI. At the same time, risks and benefits of antibiotic use should be weighed carefully^34^.

Our study has some limitations. First, it involved a single center with a relatively small number of cases, and there may have been a bias toward patients with less severe disease, since seriously ill patients at our hospital are often transferred to larger, better-equipped hospitals. The retrospective nature of our study means that we cannot draw conclusions about causal relationships linking PAUD with other factors or prognosis. Finally, The DDD value, established by WHO, is an artificial value. It is mainly used for the comprehensive evaluation of antibacterial drugs. The guiding significance of PAUD for individual treatment plans needs to be further explored.

## Conclusions

Our results suggest that PAUD is closely related to the prognosis of intracerebral hemorrhage patients with SAI. Clinical application of antibiotics in SAI patients should be considered carefully to avoid an increase in risk of in-hospital mortality. Our findings should be verified and extended in large, prospective studies.

## Data Availability

The datasets used and/or analyzed during the current study are available from the corresponding author on reasonable request.
